# How and why women’s life histories and homelessness services generate heterogeneous recovery outcomes in Barcelona: a co-created realist evaluation protocol

**DOI:** 10.3389/fpubh.2026.1939870

**Published:** 2026-10-28

**Authors:** Montserrat López-Fernández, Lucinda Cash-Gibson, Joana Morrison, Astrid Escrig Piñol, Philippe Mortier, Giuliana Raffaella Longworth

**Affiliations:** 1 Hospital del Mar Research Institute, Barcelona, Spain; 2 Departament de Medicina i Ciencies de la Vida, Universitat Pompeu Fabra, Barcelona, Spain; 3 Research Group on Health Inequalities, Environment, and Employment Conditions (GREDS-EMCONET), Department of Political and Social Sciences, Universitat Pompeu Fabra, Barcelona, Spain; 4 Universitat Pompeu Fabra- Barcelona School of Management, Barcelona, Spain; 5 JHU-UPF Public Policy Center (JHU-UPF PPC), Universitat Pompeu Fabra (UPF), UPF Barcelona School of Management (UPF-BSM), Barcelona, Spain; 6 Escola Superior d’Infermeria del Mar, Barcelona, Spain; 7 Centro de Investigación Biomédica en Red de Epidemiología y Salud Pública, Instituto de Salud Carlos III (CIBERESP, ISCIII), Madrid, Spain; 8 Glasgow Caledonian University School of Health and Life Sciences, Glasgow, United Kingdom

**Keywords:** co-creation, community health services, homelessness, personal recovery, psychological trauma, realist evaluation, systemic inequalities, women

## Abstract

**Introduction:**

Homelessness is rising in Spain, particularly in Barcelona, where women are the fastest-growing subgroup, accounting for approximately 30% of service places. Yet services have historically been designed by default for men. Women’s pathways into homelessness are shaped by individual, social, and structural determinants which increase trauma exposure and make this population highly heterogeneous. While evidence highlights the need for gender- and trauma-informed services adapted to different subgroups, there remains limited systematic understanding of how women’s life experiences influence their engagement with and outcomes from homelessness services. This study aims to understand how and why women’s life experiences and determinants interact with homelessness service and intervention elements to activate or inhibit mechanisms (e.g., personal strengths) leading to different recovery outcomes (e.g., identity), thereby informing recommendations on how services can better support person-centered recovery of women.

**Methods:**

This study protocol describes a realist evaluation that will combine a realist review with realist interviews, using co-creation methodologies involving homelessness service users and providers, through Community Advisory Committees, in Barcelona. An initial programme theory will be co-designed in parallel with literature screening and appraisal. Data will be analyzed to extract context–mechanism–outcome configurations explaining how women’s experiences and determinants generate different outcomes when interacting with service/intervention components. Co-refined programme theories will subsequently be tested through interviews and focus groups, with data analyzed retroductively and co-interpreted using different theoretical lenses and approaches such as the Social Determinants of Health, Trauma Theory, Trauma-Informed Care and the Power Threat Meaning Framework.

**Discussion:**

The study integrates realist evaluation with co-creation methodologies, enabling the development of evidence-based, context-sensitive middle-range programme theories while prioritizing the voices of both professionals and people with lived experience in the knowledge-building process, aiming for epistemic justice. Co-created causal, feminist and trauma-informed explanatory theories linking women’s experiences, mechanisms of change and intervention outcomes in Barcelona are intended to serve as a foundation for the co-production of actionable service guidelines for homelessness, as well as policy recommendations. Findings will be disseminated to both the academic and general public and will include the participation of co-researcher stakeholders for this purpose.

## 1 Introduction

Homelessness is an increasingly pressing social and public health challenge, impacting the lives and health of individuals and communities across the globe (1). It includes situations of rooflessness, houselessness, and insecure or inadequate housing, according to the European Typology of Homelessness and Housing Exclusion (ETHOS) (2). In Europe, the rate of people experiencing homelessness has increased by 70% during the last decade (3). In Spain, women represent the fastest-growing subgroup within the homeless population (4). Approximately 29% of individuals accommodated in services within the *Xarxa d’Atenció a Persones Sense Llar* — a network of organisations, including public administration services, that provide support to people experiencing homelessness in the city of Barcelona—were women in 2024 (5). By the end of 2025, Barcelona had reached a record high in the number of people experiencing homelessness: approximately 2,000 individuals were counted within ETHOS category 1 (rooflessness) alone, representing a 43% increase in just two years (6).

The profile of women experiencing homelessness is highly heterogeneous, shaped by the interactions of personal histories with structural and social determinants such as poverty, lack of affordable housing, migration, stigma, and inadequate social support (7). These determinants increase exposure to trauma through loss of stable housing, the uncertainty of meeting basic needs, and increased exposure to physical and sexual violence (8, 9). Additionally, individuals experiencing homelessness have a high prevalence of adverse childhood experiences (ACEs), which is particularly pronounced among women and contributes to long-term mental health difficulties (10–12). Furthermore, gender-based violence—including intimate partner violence and sexual violence—is a common driver of homelessness among women (13). These life determinants and experiences have profound emotional, cognitive, and physical impacts, making a trauma-informed approach essential when supporting this population and positioning personal recovery as a critical component of care. Within this study, recovery is understood as “a *way of living a satisfying, hopeful, and meaningful life, as defined by the individual, even within the fluctuations and limitations caused by states of distress”* (14).

However, homelessness services have historically been designed around male experiences and frequently lack systematically embedded gender- and trauma-informed practices (15). As a result, women may experience stigma, mistrust, and feelings of unsafety within services, which can hinder engagement, reduce the effectiveness of support, and further contribute to revictimization (15–17). In Barcelona, findings reflect similar patterns and further highlight that services often lack a holistic, integrated approach, as well as a meaningful incorporation of women’s life histories into intervention practices, resulting in fragmented responses that fall short of addressing the complexity of their needs (18, 19).

Hence, the acceptability and effectiveness of interventions might not only depend on what is delivered but also on how it is delivered—for instance, through peer support, anti-oppressive practices, autonomy-enhancing strategies (17). This aligns with evidence showing that subgroups of people experiencing homelessness (based on sociodemographic factors, health, trauma, and resilience) respond differently to a Housing First intervention (20).

Addressing personal recovery therefore requires moving beyond a ‘one-size-fits-all’ model towards person-centered, gender- and trauma-informed services. Despite growing recognition, little is known about how women’s individual life histories influence engagement with and outcomes from homelessness interventions or services. The study seeks to address this knowledge gap in the context of Barcelona.

### 1.1 Aims and objectives

This paper presents a study protocol of a co-created realist evaluation designed to (i) examine how different life events or determinants when interacting with service components (context) generate diverse outcomes of interventions or services for women experiencing homelessness (i.e., personal recovery such as connectedness or hope); (ii) identify the mechanisms that are activated or inhibited to generate the intervention/service outcomes in specific contexts; and (iii) analyze patterns between different combinations of contextual conditions, activated or inhibited mechanisms, and intervention/service outcomes.

In broader terms, the project aims to co-create an explanatory middle-range theory by first synthesizing scientific and grey literature; second, testing and adapting these theories locally through realist interviews with service users and providers in Barcelona; and finally, co-producing actionable service guidelines and policy recommendations.

Although centered on the context of Barcelona, the overall findings aim to inform the design of tailored, recovery-oriented homeless services for women in similar high-income urban settings.

## 2 Methods and analysis

The study will use a transdisciplinary approach, integrating realist evaluation, co-creation methodologies, a realist review of literature and realist interviews to achieve its research objectives Figure 1. Multidisciplinary scientific knowledge (from public health, social sciences, critical realism, psychology, health services research), and non-academic knowledge will be integrated through the study. Within this design, co-creation plays a central role: it strengthens mechanism identification—including hidden or hard-to-surface mechanisms—supports validation of contextual assumptions, helps identify unintended consequences, and ensures that emerging theories reflect lived realities rather than researcher-driven abstractions.

**Figure 1:**
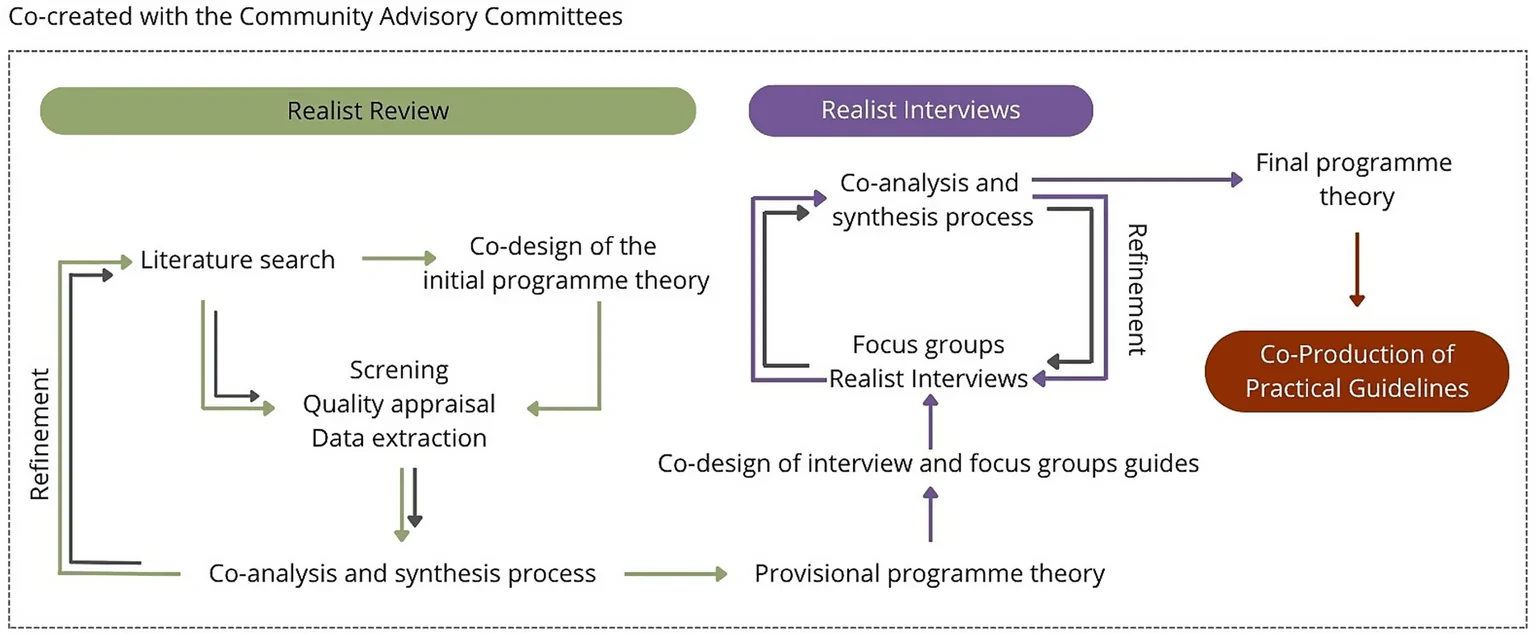
Overview of the research process.

A Gender lens is applied transversally through the research design/all phases of the research and its outputs. The study focuses exclusively on women (cis and trans), recognizing that trajectories to homelessness, experiences within it, and associated needs are gendered and require different approaches than those used for men. An intersectional feminist perspective highlights how gender interacts with other social identities (race, class, ability, age), shaping experiences within interconnected systems of oppression (21). The formulation of determinants or life experiences as a contextual situation to which services must respond is grounded in this intersectional perspective.

The research team acknowledges that researchers’ social contexts influence knowledge. Five white women and a white man with higher education and privileges, the team also includes LGBTQIA+ members and those close to trans experiences, enhancing awareness of gender diversity. Reflecting on our socio-educational privileges, we use co-creation with Community Advisory Committees (CACs) of women with lived experience and service providers to address power imbalances. Continuous reflexivity during data collection, interpretation, and reporting will help critically assess our assumptions (see Figure 1).

### 2.1 Realist evaluation

Realist evaluations are grounded in the realist philosophy of science, which posits that programmes (policies, services or interventions) are theories put into practice. Thus, these are embedded within open social systems that are dynamic and susceptible to change. For this reason, realist evaluation recognizes that causation is contingent on specific social, political, and economic contexts (22).

This approach is particularly well-suited to examining complex social phenomena involving human decisions and behaviors (22), such as the diversity and complexity of individual life trajectories. Also, as an approach it fits the project scope as it allows for the integration of the values, preferences, and lived experiences of people experiencing homelessness, which are essential for understanding intervention relevance, acceptability, and adherence (17). Realist evaluations offer an analytical framework for examining the causal mechanisms that make up programme theories, articulated through Context-Mechanism-Outcome (CMO) configurations. In realist terms, Context (C) refers to the conditions in which programmes are implemented—those aspects that influence how mechanisms operate. Examples include women’s lived experiences prior to homelessness such as gender-based violence, childhood trauma, being a transgender woman, or forced migration and homelessness service elements such as access in general, access to women-only spaces or permanent staff. Mechanisms (M) are the underlying causal processes activated or inhibited by the programme that leads to change; these are often hidden and drive observable patterns—for instance, shifts in meaning-making or the activation of personal strengths. Outcomes (O) are the intended or unintended consequences of the service or intervention, such as a strengthened sense of belonging, increased sense of agency, or improved self-image (23).

The types of interventions and services included in the study are not restricted, as the study is exploratory to ensure service inclusivity. The realist evaluation will be conducted in two phases: Phase 1 will consist of a realist review of the grey and scientific literature to co-develop potential programme theories (PTs) that explain the research question. Secondly, realist interviews with both service providers and women using these services, to delve into context and mechanisms not addressed in the literature and adapt the PTs to the specific context of Barcelona.

The process will be conducted by adapting Wong et al.’s process for realist evaluation and adhering to RAMESES quality and reporting standards (24).

#### 2.1.1 Co-creation

We will embed co-creation throughout the design and implementation of the realist evaluation. Integrating the community perspective ensures research priorities and outcomes align with local concerns (25, 26). The realist evaluation involves the active engagement of women with lived experience and professionals through CACs: one composed of women with previous lived experience and professionals from health and social sectors based in Barcelona; and a second one composed of women with current lived experience. The CACs will be involved in the development and refinement of the realist evaluation outputs, ensuring their relevance to real-world settings.

Collaborating organizations were recruited through convenience sampling. A range of local organizations involved in services for people experiencing homelessness, including organizations with specific expertise in women’s homelessness, were contacted, and those willing and able to participate were recruited. Recruitment to the CAC of women with lived experience of homelessness will be coordinated through a single participating organization, allowing meetings to be held at the same location and reducing travel barriers. It will aim to ensure diverse profiles, particularly in terms of background, ethnicity, gender identity, and sexual orientation. Potential participants will receive accessible information about the project and the implications of participation and, if interested, will be contacted by the research team to address any questions and obtain informed consent. The recruitment strategy and the organization and format of the co-creation sessions will first be discussed with professionals and subsequently refined in collaboration with the women recruited to participate, ensuring that the process is responsive to participants’ preferences, needs, and practical considerations.

Both CACs will participate independently throughout the different stages of the co-creation process, using methods tailored to the needs, preferences, and capacities of each participant group. To minimize power imbalances, several strategies will be implemented: (i) conducting separate sessions for professionals and women with lived experience; (ii) employing facilitation techniques that encourage equitable participation (e.g., written activities and object-based turn-taking); and (iii) adapting all research materials and discussions to plain language.

It will be designed and delivered in accordance with feminist and trauma-informed principles (27–29). This includes promoting the inclusion of participants with diverse gender identities and ethnic backgrounds, fostering trust and psychological safety, and developing a collaboratively agreed crisis protocol to be implemented if required.

The co-creation process will be evaluated using the PROSECO framework (30), with different components assessed for women with lived experience and professionals.

#### 2.1.2 Phase 1: Realist review

Also grounded in realist philosophy, the *realist review* is a theory-driven approach to research that seeks to explain how, why, for whom, and in what circumstances social programs work by elaborating and testing programme theories against literature. Realist reviews are characterized by a flexible and iterative structure, incorporate stakeholder consultations and draw from a wide range of sources using different methodological approaches (31). The review will follow RAMESES standards (32) and adopt a theory-driven approach while also integrating CACs’ input to ensure PTs are relevant and applicable.

The realist review will comprise six different steps: 1.1 Search strategy development; 1.2 Literature selection and appraisal; 1.3 Co-design of the Initial Programme Theory; 1.4 Data extraction; 1.5 Identification of formal theories and 1.6 Co-analysis and synthesis process with the CACs.

##### 2.1.2.1 Step 1.1: Search strategy development

To support the development of PTs and CMO configurations, both scientific and grey literature will be considered. Only studies published in English, Catalan, or Spanish will be included. The search will incorporate articles published from 2010 onward, marking the beginning of a new wave of research that incorporates a stronger gender perspective in the analysis of homelessness (16), and will be conducted across different databases, including PubMed, Social Science Database and PsychINFO. These databases were selected to provide complementary coverage of public health and healthcare, psychological processes and trauma, and social services, gender, and exclusion. Given the iterative and theory driven nature of realist reviews. This combination was considered sufficient to achieve conceptual saturation and identify the literature needed to identify potential mechanisms and refine emerging PTs. The search terms are based on key terminology used for gender, residential exclusion and the interventional/service block. Table 1 outlines the main concepts and keywords chosen to capture studies that explicitly address programs related to women’s homelessness.

**Table 1:** Search strategy terms.

Search strategy terms
(“Women”[TIAB]) OR (“Female”[TIAB]) OR (“Gender” [TIAB])
(“Ill-Housed Persons”[Mesh]) OR (“Homeless*”[TIAB]) OR (“Unhoused”[TIAB]) OR (“Houseless*”[TIAB]) OR (“rough sleep*”[TIAB]) OR (“roofless*”[TIAB]) OR (temporary accommodation*[TIAB]) OR (temporary hous*[TIAB])
(“Crisis Intervention”[Mesh]) OR (“Psychosocial Intervention”[Mesh]) OR (“Early Intervention, Educational”[Mesh]) OR (“Early Medical Intervention”[Mesh]) OR (“Health Services Needs and Demand”[Mesh]) OR (“Needs Assessment”[Mesh]) OR (“Social Work”[Mesh]) OR (“Adherence Interventions”[Mesh]) OR (“interv*”[TIAB]) OR (“service*”[TIAB]) OR (“support*”[TIAB]) OR (“need*”[TIAB]) OR (“therapy”[TIAB]) OR (“treatment*”[TIAB])
Filters: Adult: 19 + years.

Search terms were structured around three core concepts: women, homelessness, and interventions/services. Boolean operator “AND” were used to combine terms. Filters included adults (19 + years).

Grey literature search will be conducted on the webpages from organizations working with the studied population, as for example, Homeless Link, participant organizations [some of them belonging to *Xarxa d’Atenció a Persones Sense Llar* (XASPLL)], European Federation of National Organizations working with the Homeless (FEANTSA) or The Homeless Hub. Editorials, opinion pieces, practitioner communications, and other relevant materials will also be considered if they offer useful insights.

Additional literature will also be identified through snowballing of relevant papers (seed references) and screening of potentially relevant articles retained from the initial search. These references will be recorded in Covidence and reported in the PRISMA flow diagram.

##### 2.1.2.2 Step 1.2: Literature selection and appraisal

Relevant sources will be screened according to the following criteria: (i) studies involving only women aged 19 or older experiencing homelessness or analyzing women as a subgroup; (ii) studies that address life events or determinants in the interventions or services for women experiencing homelessness; and (iii) studies exploring women’s experiences with social and health interventions or services, interventional studies or case studies on interventions; (iv) studies conducted in high-income countries will be included, to ensure contextual relevance to Barcelona. Studies will be excluded if they: (i) focus exclusively on specific social groups such as veterans, prisoners, or on very specific moments in time (such as during the COVID-19 pandemic); or (ii) involve purely biomedical or pregnancy focused interventions. Table 2 summarizes the literature search.

**Table 2:** Formal literature search.

Formal literature search	
Intervention	Social and health interventions or services aimed at women experiencing homelessness
Setting	Any setting providing services or interventions for people experiencing homelessness, including shelters, outreach programmes, healthcare facilities, and community-based organisations
Participants	Adult women (≥19 years) experiencing homelessness from ETHOS categories 1, 2, 3 and 4 Professionals delivering services Women with past experiences of homelessness
Study design	All study designs
Outcome	Any intervention/service outcomes, prioritising recovery outcomes (e.g., connectedness, hope, identity, purpose, empowerment)
Limits	2010-present. Only studies from high-income countries written in Spanish, Catalan and English will be included.
Databases	PubMed, Social Science Database and PsychINFO. Grey literature from associations part of XASPLL, Single Homelessness Project, Canadian Observatory on Homelessness, Homelessness Australia, FEANTSA and The Homeless Hub
Inclusion criteria	Studies with women (≥19) experiencing homelessness, addressing life factors or events, and exploring their experiences with social or health interventions or explaining how or why these worked.
Exclusion criteria	Studies focused exclusively on specific social groups such as veterans, prisoners or pregnant women, or in very specific moments in time (such as during the COVID-19 pandemic or involve purely biomedical interventions).

Covidence, a systematic reviews management software, will be used to manage and screen the studies identified in the review, supporting systematic data extraction and quality appraisal (33).

Titles and abstracts will be screened by one researcher, and a 10% random subsample of the documents retrieved from searching will be assessed independently by three researchers for quality control. The full texts of potentially relevant publications will be screened by three or more researchers. Any disagreements that are not resolved among the three researchers will be resolved through majority vote within the research team.

At the point of inclusion, relevance, richness and rigor will be assessed in accordance with the suggestions described by Dada et al. (34) and the Initial Programme Theory (IPT; see Step 1.3). Judgements about rigor will be made at the level of the included data and at the PT’s level.

##### 2.1.2.3 Step 1.3: Co-design of the initial programme theory

In parallel to the search strategy, a candidate IPT was defined by the research team. This IPT serves as a starting point to guide the search, define the inclusion and exclusion criteria and prepare the richness and rigor templates. This IPT was based on current literature on the topic and different disciplinary theories and approaches, including the Power Threat Meaning Framework (PTMF), the Social Determinants of Health (SDOH), and Judith Herman’s Trauma Theory, but also suggestions from Trauma-Informed Care (TIC) (35–39). At this stage, the IPT is intentionally broad and exploratory. This openness is deliberate to allow the identification of “unknown unknowns,” unanticipated contextual factors and mechanisms that may be essential to explaining how, for whom, and under what conditions services work:

If homelessness services recognize and adequately consider the life experiences and broader social determinants of women experiencing homelessness in their design and implementation, then will increase their engagement with and outcomes from these services, and improve their personal recovery, because this will activate the psychological, social and resilience processes necessary for women’s recovery (such as perception of safety, restore agency, feeling empowered, prevent traumatization, activate resilience).

First, the SDOH situate the material and social constraints and exposures that create objective threats and limit the opportunities and resources necessary for personal recovery from experiences of homelessness (36, 37); improvements in these determinants are therefore expected to precede and condition psychological and behavioral mechanisms central to the IPT.

Second, Herman’s (38) work conceptualizes prolonged interpersonal violence as disrupting both intrapsychic and relational functioning. She introduced the complex post-traumatic stress disorder to characterize the cluster of symptoms seen after prolonged, inescapable trauma (situations she describes as involving captivity, coercive control, and enforced dependency), which foregrounds the relational and developmental dimensions of traumatic harm. She further emphasizes the roles of memory and meaning-making, proposes a staged model of recovery, and highlights the political nature of trauma and its silencing.

Third and consequently, TIC operationalizes trauma theory defining service-level principles (trauma awareness, prioritization of safety, promotion of choice/control, strengths orientation) that map directly onto the IPT’s proposed mechanisms (perceived safety, restored agency, feeling empowered, prevention of traumatization, activation of resilience) (39). These two theories inform the IPT by highlighting the importance of recognizing women’s histories within homelessness services to enhance engagement and outcomes, and by identifying the elements that need to be enabled to achieve this (e.g., safety, agency, empowerment, and resilience).

Fourth, the PTMF helps interpret how the context where experiences of violence or exclusion happen shape the subjective meanings women give, and thus, their responses, in this case, to services. It organizes general patterns of coping and survival strategies through four interconnected domains: “Power,” “Threat,” “Meaning,” and “Threat Responses.” The framework highlights how social injustice and adverse experiences can generate psychological distress and behaviors that, while often labelled as problematic, represent adaptive responses to adversity. In doing so, it underscores that psychological wellbeing is inseparable from material, social, environmental, and cultural contexts. Importantly, the inclusion of “Power” enables an intersectional perspective (40), making visible how overlapping systems of oppression shape women’s different pathways into and out of homelessness. This approach reframes distress and so-called problematic behavior as functional responses rather than symptoms, offering a comprehensive lens to understand psychological distress in contexts of trauma and social inequality.

Afterwards, the candidate IPT will be shared with the CACs. The co-creation session will aim to refine the IPT by identifying missing components to inform the development of the data extraction template, ensuring that relevant context, mechanism and outcome configurations can be systematically extracted from the selected literature.

##### 2.1.2.4 Step 1.4: Data extraction

A data extraction template will be used to support consistency and transparency. Data extraction and data organization will be undertaken by three researchers, with a random sample of 10% independently checked by other researchers for quality control, with disagreements handled in the same way as above. The template will include descriptive characteristics of each document, along with extracted content relevant to theory development. For instance: e.g. if a woman has experienced prolonged intimate partner violence and receives trauma-informed, gender-sensitive housing support (Context), then she is more likely to engage with services and maintain housing stability (Outcome), because the intervention activates a sense of safety and trust (Mechanism). e.g., if a woman with a history of childhood care experiences enters a housing program with rigid compliance requirements (Context), then she may disengage or exit early (Outcome), because the intervention triggers feelings of control and surveillance that undermine her sense of autonomy (Mechanism).

The characteristics of the documents will be extracted into an Excel spreadsheet design in accordance with the IPT.

##### 2.1.2.5 Step 1.5: Identification of formal theories

To help make sense of the data, PTMF (35) will serve as the main analytic framework. It is particularly suitable for understanding how factors and experiences within women’s life pathways influence responses to services, as it integrates social, relational, and material contexts, emphasizes personal meaning, highlights structural inequalities, and recognizes both threats and strengths. The PTMF provides the primary lens, which encompasses trauma theories and feminist perspectives, and this will be complemented by the SDOH framework (35–38). See Table 3 for an overview of the roles of the theoretical lenses used in the realist review data analysis.

**Table 3:** Role of the different theoretical lens in the data analysis.

Theoretical lens	Function	Examples of potential mechanisms
PTMF	Explains the role of meaning in responses to adversities while including the structural/political layer on psychological distress	Changes in meaning-making, adaptative responses, perceived threats
Trauma theory and TIC	Informs on intra-psychic and relational dimensions of trauma and recovery stages. TIC operationalize this knowledge in service practices	Hyperarousal, numbing, memory, trust, safety, agency
SDOH	Emphasizes the role of structural and social context in shaping health outcomes	Material deprivation, precariousness, access to resources

Selected theories will be used as complementary analytical lenses rather than as competing theoretical frameworks. The PTMF provides a particularly integrative framework, drawing on the other theories, including feminist theory. Flexibility will be maintained to incorporate additional theories as needed for mechanisms beyond the reach of the chosen frameworks.

##### 2.1.2.6 Step 1.6: Co-analysis and synthesis process with the CACs

Qualitative data analysis will draw on methods from the realist evaluation methodological guidance (41). A retroductive logic will guide coding, treating each extracted coded text as a discrete data source coded against PT-related nodes. New patterns or contradictions will lead to the creation of child nodes to support theory refinement. Memos will be systematically attached to nodes to document interpretive decisions, context, mechanisms, outcomes, and relationships between PTs and CMOs, promoting transparency and traceability.

The CACs will engage in a structured co-interpretation process, critically examining the relevance and generating better possible explanations of the emerging CMO configurations. Abductive reasoning, which is particularly amenable to participatory inquiry, is a key phase in realist analysis. Together with retroductive reasoning, it allows for the refinement, revision, or rejection of theoretical propositions as new insights emerge (42). Thus, to refine the resulting PTs, further literature searches may be conducted on PTs from the realist review that were agreed with the CACs.

#### 2.1.3 Phase 2: Realist interviews

##### 2.1.3.1 Step 2.1: Data collection: co-designed interviews and focus groups with the CACs

Realist interviews and focus groups will be conducted with women with lived experience (WLE) and professionals to further refine the CMO configurations and initial PTs in the context of Barcelona, and elaborate new PTs, expanding on the complexity of WLE and its interactions with specific service elements. Interview questions and focus group guides will be co-designed with the CACs. Following the principles outlined by Manzano (43) and Manzano and Williams (44), interviews will be structured around a theory-driven approach, using a semi-structured guide informed by preliminary CMO configurations. The interviewer will adopt a reflexive stance and engage participants in a teacher–learner cycle, whereby emerging theories are shared and discussed collaboratively.

Participants (both women with lived experience and professionals) will be selected through theoretical purposive sampling, guided by emerging programme theories derived from the realist review (e.g., experiences of gender-based violence, being a transgender women, complex trauma, and migration histories). We will add open-ended prompts capturing legal/medical navigation, and identity-specific structural barriers for the different trajectories.

For women with lived experience, eligibility will include being aged 18–65 years and being able to participate in and complete the interview. Women whose cognitive impairment or current substance use substantially interferes with their ability to participate in the interview will be excluded. The different collaborating organizations will support the identification of participant profiles and service contexts required to refine the PT. To ensure the recruitment process is responsive to the needs and circumstances of people experiencing residential exclusion, recruitment procedures will be co-developed with the CAC.

Focus groups will be conducted with approximately 10–20 professionals, while in-depth interviews will be carried out with approximately 10–15 women with lived experience of homelessness. Focus groups with professionals will facilitate discussion of PTs drawing on their experience with different groups of women, thus being able to refine the PT for different contexts. Individual interviews with women experiencing homelessness from diverse backgrounds will provide a private setting in which participants can decide what they feel comfortable disclosing and discuss potentially distressing experiences without the presence of other participants, in line with trauma-informed research principles. In addition, the practical difficulties of coordinating group sessions with women experiencing homelessness support the use of individual interviews.

Data collection will primarily take place face-to-face, with flexibility for online formats if required. Interviews will be conducted in Spanish, Catalan, Italian, or English. Sample size will remain flexible, as is typical in realist research (43), to achieve sufficient explanatory depth and theoretical coverage of emerging CMO configurations. All interviews and focus groups will be audio-recorded using a recording device, after obtaining consent, to ensure accurate data capture. Audio files and related materials will be securely stored for 25 years, in accordance with the approved ethical protocol. Only the principal researcher and the research technician. Data will be transcribed with the assistance of Happy Scribe, a GDPR-compliant service.

##### 2.1.3.2 Step 2.2: Co-analysis and synthesis process with the CACs

Transcripts will be produced verbatim and de-identified to maintain participant anonymity. An immersive reading of all transcripts and field notes will provide contextual understanding. Data analysis will be conducted as described in step 1.6. We will apply an intersectional analytical framework to disaggregate shared gender experiences from trajectories unique to different trajectories. New CMOs emerging from the collected data will fill gaps not covered by the literature and will be used to locally adapt the PTs. The process will lead to a co-developed middle-range theory.

### 2.2 Co-production of practical guidelines

The PT will be used to examine how and why specific aspects of women’s life experiences, such as their social identities or experiences of childhood trauma, may contribute to different outcomes depending on how services and interventions are designed and implemented. This will help identify which elements of service design and implementation may contribute to different outcomes according to women’s life circumstances and contexts, and to explore the mechanisms underlying these differences.

The co-production phase will result in two main outputs: (1) a blueprint-style guide for professionals and (2) a policy recommendations document addressing aspects identified through the findings whose modification depends on policy decisions. The main output will be the professional guide. The CAC will contribute to determining the most appropriate sections, content, and format to ensure that the guide is practical and useful for professional practice. The translation of the theoretical and research findings into clinical and social practice will also be jointly agreed with the CAC.

Based on this process, an initial draft will be developed and subsequently reviewed and, where necessary, modified by the CAC until a final agreed version is reached. The CAC will also contribute to validating the language and format of the guide to ensure that it is clear, understandable, and actionable for the professionals for whom it is intended. This process will remain flexible and open to modification throughout its development, in keeping with the co-created nature of the guide.

## 3 Discussion

This study protocol outlines a novel, transdisciplinary approach to understanding how the heterogeneous life experiences and structural determinants of women experiencing homelessness in Barcelona interact with specific service components to shape personal recovery outcomes. To our knowledge, this project is a pioneering effort to systematically integrate realist evaluation with participatory co-creation methodologies under an intersectional feminist and trauma-informed framework. Through the embedded governance of the CACs that bridges the expertise of service providers and women with lived experience, this design explicitly prioritizes epistemic and testimonial justice in the knowledge-production process.

The project acknowledges that not all traumatic experiences have the same impact, nor does it seek to pathologize or individualize symptoms that may be rooted in broader structural determinants. For example, while ACEs are consistently associated with poorer mental health outcomes and homelessness trajectories, different forms, timing, severity, and chronicity of adversity may generate distinct impacts. ACEs themselves are not randomly distributed across the population, but are strongly associated with systemic inequities (45). Consequently, the PTMF provides a useful framework to understand distress and coping responses as adaptive reactions to threats posed by how power operates across people’s lives, thereby reducing pathologizing and stigmatizing interpretations and moving beyond deficit-oriented and individualized explanations.

The study integrates realist evaluation with co-creation methodologies, a combination that remains relatively underexplored in the literature. This integration enables the coupling of a theory-driven explanatory framework with participatory co-creation methodologies co-produced knowledge to develop a context-sensitive explanatory framework through retroductive data analysis (46, 47).

The integration of realist evaluation and intersectional feminism poses an apparent paradox. On the one hand, realist evaluation is grounded in critical realism, which assumes an intransitive reality—causal mechanisms exist independently of whether we know them. On the other hand, co-creation and intersectional feminist theories draw on social constructivist approaches, where knowledge, contexts, and even identities are understood as co-constructed, processual, and situated. This tension, however, is not irresolvable. Critical realism assumes epistemic relativism insofar as knowledge is fallible and transitory, thereby allowing knowledge to be situated and context-relative without denying the existence of a material reality (48). In other words, although there is a reality independent of us, our access to it is always mediated by interpretative frameworks, languages, and social positions. In this line, co-creation can play an important role in realist analysis by enriching abductive reasoning. Abduction, central to the realist paradigm, involves reinterpreting patterns through alternative theoretical lenses to generate plausible explanatory mechanisms. Co-creation, by systematically incorporating the voices of women with lived experience and practitioners, expands the range of possible explanatory resources and strengthens the construction of more plausible accounts of generative mechanisms underlying observed outcomes. Co-creation thus serves as a constructivist method in the service of a realist explanatory goal, rather than an end in itself. The refined PTs generated through this realist study will provide an explanatory foundation for the co-production of policy recommendations and actionable guidelines aimed at supporting the development of person-centered, feminist, and trauma-informed homelessness services. Findings will be disseminated to both the academic and general public and will include the participation of CAC entities for this purpose.

The design of this evaluation has some limitations arising from the realist review. First, focusing exclusively on women currently experiencing homelessness may limit conclusions about long-term recovery trajectories. Second, restricting the review to high-income settings ensures contextual relevance to Barcelona but excludes potentially informative research from low- and middle-income countries. Third, excluding homeless veterans and pregnancy-specific interventions, although justified, may omit relevant findings for specific subgroups of women. Fourth, the screening of titles and abstracts by a single researcher, with an independent quality check of a random 10% subsample by other researchers, may result in some relevant studies being missed; however, this approach is consistent with established guidance for realist reviews.

Because recruitment relies heavily on established third-sector and hospital networks, the study may inadvertently miss highly marginalized, invisible women with lived experience. This includes women suffering sexual exploitation, undocumented or displaced women who may avoid institutional contact due to mistrust or previous institutional violence by authorities.

Women who speak other languages may face barriers to full participation in the CAC or in-depth interviews, potentially limiting the capture of nuances of certain migration pathways.

The resulting PTs are intended to serve as explanatory patterns, heuristic tools—functioning as demi-regularities—rather than deterministic templates. Human behavior and recovery pathways are highly dynamic, and the protocol does not seek to reduce women’s unique trajectories into rigid, predictive formulas striving to protect individual agency.
